# Estimating cross-relaxation rates between methyl and neighboring labile proton spins in high molecular weight proteins

**DOI:** 10.1007/s10858-025-00469-8

**Published:** 2025-05-25

**Authors:** Vitali Tugarinov, G. Marius Clore

**Affiliations:** Laboratory of Chemical Physics, National Institute of Diabetes and Digestive and Kidney Diseases, National Institutes of Health, Bethesda, MD 20892-0520 USA

**Keywords:** Exchange saturation transfer, Cross relaxation, Hydrogen exchange, Methyl NMR of high-molecular-weight proteins, Water saturation

## Abstract

We show that water saturation leads to deleterious losses in sensitivity of methyl signals in selectively methyl-[^13^CH_3_]-labeled protein samples of high molecular weight proteins dissolved in H_2_O. These losses arise from efficient cross-relaxation between methyl protons and proximal labile protons in the protein structure. A phenomenological model for analysis of methyl intensity decay profiles that involves exchange saturation transfer of magnetization from localized proton spins of water to various labile groups in the protein structure that, in turn, efficiently cross-relax with protons of methyl groups, is described. Analysis of methyl intensity decay profiles with this model allows cross-relaxation rates (σ) between methyl and labile protons to be determined and permits identification of methyl sites in close proximity to labile groups in the protein structure.

Methyl groups have played a special role in NMR studies of protein structure and side-chain dynamics yielding important insights into kinetics, thermodynamics and mechanisms of a large variety of biochemical processes (Tugarinov et al. 2004; Ruschak and Kay 2010; Rosenzweig and Kay 2014; Schütz and Sprangers 2020). Typically, NMR experiments targeting methyl groups of high molecular weight proteins are applied to protein samples that are selectively isotopically labeled with ^13^CH_3_ methyl groups on a highly deuterated background (Tugarinov et al. 2006). Although for many NMR experiments that target methyl groups, the isotopically labeled protein samples may be prepared in D_2_O solvent, oftentimes they are dissolved in H_2_O as a matter of convenience – for example, if the same sample is planned to be used for NMR applications involving backbone ^1^H-^15^N amide groups. In addition, H_2_O is ~ 20% less viscous than D_2_O (Cho et al. 1999), resulting in a lower global rotational correlation time, τ_C_, of the protein of interest. When selectively [^13^CH_3_]-methyl-labeled proteins are dissolved in H_2_O, however, problems may arise with water suppression in heteronuclear multiple-quantum coherence (HMQC)-based NMR experiments (Mueller 1979; Bax et al. 1983; Tugarinov et al. 2003). As methyl hydrogens do not exchange with water, it may be tempting to achieve satisfactory water suppression by saturating the water signal with a weak radio-frequency (RF) field.

Here we demonstrate that water saturation leads to deleterious losses in sensitivity of methyl signals in selectively methyl-[^13^CH_3_]-labeled protein samples of high molecular weight proteins dissolved in H_2_O. We show that even if water is saturated with a relatively weak RF field strength (< 50 Hz), the decrease in observed methyl signal intensities may be very substantial (up to a factor of 4) in high molecular weight proteins. These losses in sensitivity are traced to efficient cross-relaxation between methyl protons and the proximal labile protons in the protein structure that are in moderately fast exchange with (saturated) water. We describe a phenomenological model for analysis of methyl intensity decay profiles that involves exchange saturation transfer of magnetization from localized water proton spins to various labile groups in the protein that can, in turn, cross-relax efficiently with methyl protons. This model allows for the determination of cross-relaxation rates (σ) between methyl and labile proton spins and the identification of methyl sites in close proximity to one or more labile protons in the protein structure.

The detrimental effects of water saturation are demonstrated on the {U-[^15^N,^2^H]; Ileδ1-[^13^CH_3_]}-labeled sample of an 82-kDa enzyme Malate Synthase G (MSG) at 5 °C (τ_C_ ~ 90 ns) and 30 °C (τ_C_ ~ 45 ns) using the recently developed gradient-selected delayed decoupling ^1^H-^13^C HMQC experiments, dd-(^1^H-^13^C)-HMQC (Bolik-Coulon et al. 2023; Ahmed et al. 2024), immediately preceded by a 2.5 s-long water saturation period. Although the gradient selection of multiple-quantum ^1^H-^13^C coherences leads to a √2 loss of sensitivity compared to amplitude-modulated experiments, gradient selection is indispensible for ensuring high quality 2D (^1^H-^13^C)-HMQC correlation spectra irrespective of the strength of the water saturation field, *B*_1_, employed. For Ileδ1 methyl groups of MSG, the gradient selected dd-(^1^H-^13^C)-HMQC experiment (Ahmed et al. 2024) provides sensitivity gains of 33 ± 15% and 12 ± 9% at 5 °C and 30 °C, respectively, compared to the same experiments without delayed decoupling.

Figure 1 shows the decay profiles of methyl peak intensities typically obtained for selectively [^13^CH_3_]-labeled and otherwise deuterated samples of ubiquitin at 5 °C (τ_C_ ~ 9 ns; shown in green), and MSG at 5 °C (τ_C_ ~ 90 ns; shown in red) and 30 °C (τ_C_ ~ 45 ns; shown in blue) as a function of the saturation field strength *B*_1_. In all cases, the intensities are normalized to those obtained without water saturation (*B*_1_ = 0). The profiles have a characteristic biphasic shape, with the steep decrease in signal intensity associated with the initial phase of the curve (up to *B*_1_ ~ 25 Hz) clearly dependent on τ_C_. As we show below, the extent of this initial drop in intensity is exquisitely sensitive to cross-relaxation rates (σ) between methyl protons and proximal labile hydrogens, but also depends on: (1) longitudinal relaxation rates (*R*_1_) of both methyl and labile protons, and (2) the rate of exchange of labile hydrogens with water (*k*_*ex*_). The second phase of each curve is characterized by a monotonic decay of signal intensity that is primarily determined by transverse relaxation rates (*R*_2_) of both methyl and labile ^1^H spins. We note that this monotonic decay may be contrasted to the genuine steady state reached in heteronuclear ‘steady-state’ NOE experiments for ^1^H-^15^N/^1^H-^13^C spin-systems, where the magnetization of a heteronucleus (^15^N/^13^C) is not directly perturbed by ^1^H saturation.

**Fig. 1:**
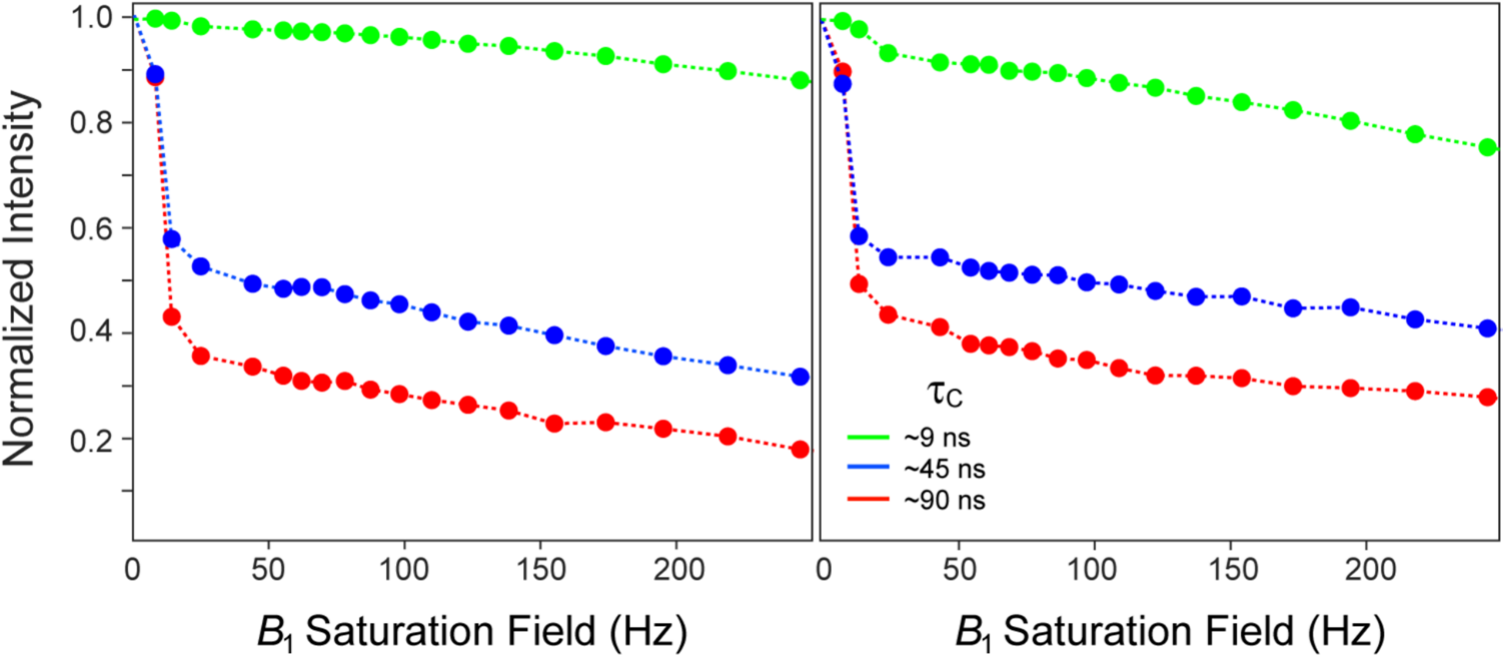
Typical intensity decay curves obtained for selectively [^13^CH_3_]-labeled and otherwise deuterated protein samples with varying rotational correlation times, *τ*_C_, as a function of the saturation field strength *B*_1_. Examples are drawn from Leu^67^ δ2 (*left panel*) and Leu^50^ δ2 (*right panel*) methyl groups of the 7.5-kDa protein ubiquitin (green circles, *τ*_C_ ~ 9 ns), and Ile^200^ δ1 (*left panel*) and Ile^260^ δ1 (*right panel*) methyl groups of the 82-kDa Malate Synthase G (MSG) at 30 ºC (blue circles, *τ*_C_ ~ 45 ns) and 5 ºC (red circles, *τ*_C_ ~ 90 ns). The 0.5 mM sample of {U-[^15^N, ^2^H]; Ileδ1-[^13^CH_3_]}-labeled MSG was dissolved in a buffer containing 90% H_2_O/ 10% D_2_O (v/v), 20 mM sodium phosphate (pH 7.0), 5 mM MgCl_2_ and 0.05% of NaN_3_. The 1.3 mM {U-[^15^N, ^2^H]; Ileδ^1^-[^13^CH_3_]; Leu,Val-[^13^CH_3_,^12^CD_3_]}-labeled sample of ubiquitin was dissolved in a buffer containing 90% H_2_O/10% D_2_O (v/v), 20 mM sodium phosphate (pH 7.0) and 50 mM NaCl. The data were obtained using either 2D gradient-selected dd-(^1^H-^13^C)-HMQC experiment (Ahmed et al. 2024) (for MSG) or a gradient-selected 2D (^1^H-^13^C)-HMQC (for ubiquitin) immediately preceded by a 2.5 s long ^1^H continuous wave (CW) saturation at the frequency of the water signal. All data were recorded on a 600 MHz AVANCE HD Bruker spectrometer equipped with a triple-axis (*x*, *y*, *z*) gradient cryogenic probe. The connecting dashed lines do not represent best-fits and are shown merely to guide the eye

The labile hydrogens in fast exchange may include non-hydrogen-bonded backbone amides (Wüthrich 1986; Grzesiek and Bax 1993); the amides of Asn and Gln side chains (Wüthrich 1986; Klein et al. 2019); hydroxyl groups of Ser, Thr and Tyr (Liepinsh et al. 1992; Takeda et al. 2011, 2009); Cys thiol groups (Takeda et al. 2010); guanidinium groups of Arg (Mackenzie and Hansen 2019); and amino groups of Lys side-chains (Iwahara et al. 2007). The abundance of labile ^1^H spins with unknown relaxation properties, chemical shifts and hydrogen exchange rates in the vicinity of each methyl site, makes rigorous modeling of the intensity decay profiles impossible. Below we describe a phenomenological, semi-quantitative approach that was adopted for analysis of intensity decay profiles with the primary focus on determination of the cross-relaxation rates σ between methyl and labile ^1^H spins. The minimalistic model that satisfies experimental intensity decay profiles, is comprised of three distinct sites: (1) methyl protons (labeled with ‘M’ superscripts), (2) labile protons (superscript ‘H’) that cross-relax with methyl protons and, at the same time, are involved in hydrogen exchange with (3) protons of ‘localized’ water molecules (superscript ‘W’). The set of Bloch-McConnell equations (McConnell 1958; Helgstrand et al. 2000) describing the evolution of magnetization of this 3-site system is given by,

1 $$\frac{d}{dt}\left[ {\begin{array}{*{20}c} E \\ {I_{x}^{{\text{M}}} } \\ {I_{y}^{{\text{M}}} } \\ {I_{z}^{{\text{M}}} } \\ {I_{x}^{{\text{H}}} } \\ {I_{y}^{{\text{H}}} } \\ {I_{z}^{{\text{H}}} } \\ {I_{x}^{{\text{W}}} } \\ {I_{y}^{{\text{W}}} } \\ {I_{z}^{{\text{W}}} } \\ \end{array} } \right] = - \left[ {\begin{array}{*{20}c} 0 & 0 & 0 & 0 & 0 & 0 & 0 & 0 & 0 & 0 \\ 0 & {R_{2}^{{\text{M}}} } & {\Omega^{{\text{M}}} } & 0 & {\sigma_{R} } & 0 & 0 & 0 & 0 & 0 \\ 0 & { - \Omega^{{\text{M}}} } & {R_{2}^{{\text{M}}} } & {\omega_{1} } & 0 & {\sigma_{R} } & 0 & 0 & 0 & 0 \\ {\Theta^{{\text{M}}} } & 0 & { - \omega_{1} } & {R_{1}^{{\text{M}}} } & 0 & 0 & \sigma & 0 & 0 & 0 \\ 0 & {\sigma_{R} /3} & 0 & 0 & {R_{{2}}^{{\text{H}}} + k_{ex} /2} & {\Omega^{{\text{H}}} } & 0 & { - k_{ex} /2} & 0 & 0 \\ 0 & 0 & {\sigma_{R} /3} & 0 & { - \Omega^{{\text{H}}} } & {R_{{2}}^{{\text{H}}} + k_{ex} /2} & {\omega_{1} } & 0 & { - k_{ex} /2} & 0 \\ {\Theta^{{\text{H}}} } & 0 & 0 & {\sigma /3} & 0 & { - \omega_{1} } & {R_{1}^{{\text{H}}} + k_{ex} /2} & 0 & 0 & { - k_{ex} /2} \\ 0 & 0 & 0 & 0 & 0 & 0 & 0 & {R_{2}^{{\text{W}}} } & {\Omega^{{\text{W}}} } & 0 \\ 0 & 0 & 0 & 0 & 0 & 0 & 0 & { - \Omega^{{\text{W}}} } & {R_{2}^{{\text{W}}} } & {\omega_{1} } \\ {\Theta^{{\text{W}}} } & 0 & 0 & 0 & 0 & 0 & 0 & 0 & { - \omega_{1} } & {R_{1}^{{\text{W}}} } \\ \end{array} } \right]\left[ {\begin{array}{*{20}c} E \\ {I_{x}^{{\text{M}}} } \\ {I_{y}^{{\text{M}}} } \\ {I_{z}^{{\text{M}}} } \\ {I_{x}^{{\text{H}}} } \\ {I_{y}^{{\text{H}}} } \\ {I_{z}^{{\text{H}}} } \\ {I_{x}^{{\text{W}}} } \\ {I_{y}^{{\text{W}}} } \\ {I_{z}^{{\text{W}}} } \\ \end{array} } \right]$$

where $I_{j}^{{\text{K}}}$ represents the deviation of the component *j* $\in${*x*, *y*, *z*} of the angular momentum of a nuclear spin for state K $\in${‘M’; ‘H’; ‘W’} from thermal equilibrium; $R_{1}^{{\text{K}}}$ and $R_{2}^{{\text{K}}}$ are longitudinal and transverse relaxation rates, respectively, of state K; Ω^K^ are frequency offsets of each state K from the frequency of the saturation field *B*_1_; *ω*_1_ is strength of the continuous-wave (CW) saturation field *B*_1_ (applied along the *x*-axis; rad/s); σ is longitudinal cross-relaxation rate between methyl protons ‘M’ and the labile proton ‘H’; σ_*R*_ is transverse cross-relaxation rate (ROE) between the sites ‘M’ and ‘H’, assumed, in the macromolecular limit, equal to, σ_*R*_ = -2σ; *k*_*ex*_ is a phenomenological rate constant that couples the evolution of labile protons with that of ‘localized’ water molecules (describing hydrogen exchange between protons ‘H’ and ‘W’); $\Theta^{{\text{M}}} = - R_{1}^{{\text{M}}} - \sigma$; $\Theta^{{\text{H}}} = - R_{1}^{{\text{H}}} - \sigma /3$; $\Theta^{{\text{W}}} = - R_{1}^{{\text{W}}}$; *E* is unity; the initial conditions (at the start of the saturation period) are: *I*_0_ = [1 0 0 0 0 0 0 0 0 0 0 0 1]^*T*^, with ‘*T*’ denoting transposition. Note that the rate of cross-relaxation of the three magnetically equivalent methyl protons ‘M’ with labile protons ‘H’ is threefold larger than that between ^1^H spins in a ^1^H-^1^H spin-pair and, in the macromolecular limit, given by, $\sigma = - \left( {3/10} \right)\left( {\mu_{0} /4\pi } \right)^{2} (\gamma_{H}^{4} \hbar {}^{2}S^{2} \tau_{C} /r_{{^{hh} }}^{6} )$, where γ_H_ is the gyromagnetic ratio of ^1^H spins, μ_0_, the vacuum permeability constant, *r*_*hh*_ is the (< *r*^−3^ >)^−1/3^-averaged distance between methyl protons and protons ‘H’; and *S* is the order parameter of the ‘M’–‘H’ interaction.

The temporal changes in the value of $I_{z}^{{\text{M}}}$, calculated by integrating Eq. (1), were tracked as a function of the strength of the saturation field, *ω*_1_, at several offsets from the water resonance frequency, Ω^W^. The set of local (site-specific) variable parameters of the combined non-linear fit of intensity decays for all Ω^W^ comprised: {σ; *k*_*ex*_; Ω^H^}, while the rates $R_{1}^{{\text{W}}}$ and $R_{2}^{{\text{W}}}$ were used as global parameters (shared between all methyl sites). The transfer of magnetization from (saturated) water to labile proton spins via hydrogen exchange in the model of Eq. (1) is reminiscent of ^1^H Chemical Exchange Saturation Transfer (CEST) experiments (Forsen and Hoffman 1963; Hoffman and Forsen 1966; Yuwen et al. 2017b, 2017a; Yuwen and Kay 2017)—particularly, their implementation for systems with *a-priori* known chemical shifts of the exchanging states (Ramanujam et al. 2019), with the important difference that methyl ^1^H spins are coupled to the neighboring labile ^1^H spins through dipolar cross-relaxation. As a consequence, the identification of proximal labile protons (Guivel-Scharen et al. 1998; Martinho et al. 2023) is indirect and based on the extracted values of cross-relaxation rates *σ*.

Hydrogen exchange of labile protons ‘H’ with water in the model of Eq. (1) involves water molecules that are spatially ‘localized’ (such as, for example, water molecules of the protein hydration shell in close proximity to labile protons (Van-Quynh et al. 2003; Bonechi et al. 2019) or those forming transient hydrogen bonds with the latter (Persson and Halle 2015)) as opposed to water molecules of the bulk solvent. If exchange with the bulk solvent is considered, the reaction of hydrogen exchange would obey the kinetic relationship, *k*_H→W_[protein] = *k*_W→H_[water], where *k*_H→W_ and *k*_W→H_ are the first-order rate constants of the forward and backward exchange processes, respectively, and *k*_W→H_ would be downscaled by the ratio, [protein]/[water]. In that case, the magnetization of labile protons would essentially be un-coupled from the pool of solvent molecules. By contrast, if water molecules participating in hydrogen exchange are localized and distinguished from the bulk solvent, it may be assumed that *k*_H→W_ = *k*_W→H_ = *k*_*ex*_/2, where *k*_*ex*_ = *k*_H→W_ + *k*_W→H_. We note that the evolution of water magnetization in the model of Eq. (1) does not depend on that of labile protons ‘H’ or *k*_*ex*_, as we are not concerned with the ‘fate’ of water magnetization in the first place, while the localized water molecules are likely to be in very fast exchange with the bulk solvent—a process that is not included in the model. Also not included in the model are possible ‘direct’ dipolar interactions of methyl protons with bound (buried) water molecules (Otting 1997), or long-range dipolar couplings with the solvent (Halle 2003). Earlier studies showed that the residence time of water molecules buried in protein structures is on the order of several nanoseconds at the highest (G.M. Clore et al. 1990; G. M. Clore et al. 1994; Ernst et al. 1995; Otting 1997), and therefore such cross-relaxation is assumed to be negligible in the context of the present study. Likewise, cross-relaxation between methyl sites themselves is not accounted for in the model of Eq. (1), as methyl proton resonances are offset from the water saturation frequency by, Ω^M^ ~ − 4 ppm, and therefore inter-methyl cross-relaxation has only a minor impact on the second phase of methyl signal decay profiles (that is absorbed in the value of Ω^H^ in the fit).

The important advantage of the formulation in Eq. (1) consists in effective de-correlation of the rates σ and *k*_*ex*_, permitting robust determination of σ for each methyl site from the set of intensity decays obtained at several offsets from the water frequency, Ω^W^, largely irrespective of the assumptions about the relaxation rates of labile protons, $R_{1}^{{\text{H}}}$ and $R_{2}^{{\text{H}}}$ (which, as we describe below, can be fixed to uniform values to restrict the number of variable parameters of the fit). Figure 2 (*left panel*) shows that the sensitivity of the shape of the intensity decay profiles simulated using Eq. (1) to the value of *k*_*ex*_ is restricted to small exchange rates (up to a certain threshold of ~ 20 s^−1^). On the other hand, although the rates σ may be strongly correlated to the offsets of labile protons from saturation frequency, Ω^H^, the sensitivity of intensity decay shapes to Ω^H^ is quite low for the range of labile ^1^H chemical shifts commonly encountered in proteins (Fig. 2, *right panel*). As a result, a very minor change in the value of σ can compensate for a major change in Ω^H^. We note that the main limitation of the model in Eq. (1) derives from its reliance on the accurate values of longitudinal methyl ^1^H relaxation rates, $R_{1}^{{\text{M}}}$, which have to be measured separately using the ‘initial rate’ approximation. Even partial break-down of the ‘initial rate’ approximation would compromise the accuracy of the extracted cross-relaxation rates. On another note, the scope of applicability of the model is constrained by the rates of hydrogen exchange between the water and labile proton spins (*k*_*ex*_)—the exchange rate slower than a certain threshold value leads to convex-shaped profiles (Fig. 2, *left panel*), irrespective of the value of σ. As a result, for slowly exchanging labile protons (*k*_*ex*_ < ~ 1 s), the cross-relaxation rates become indeterminable.

**Fig. 2:**
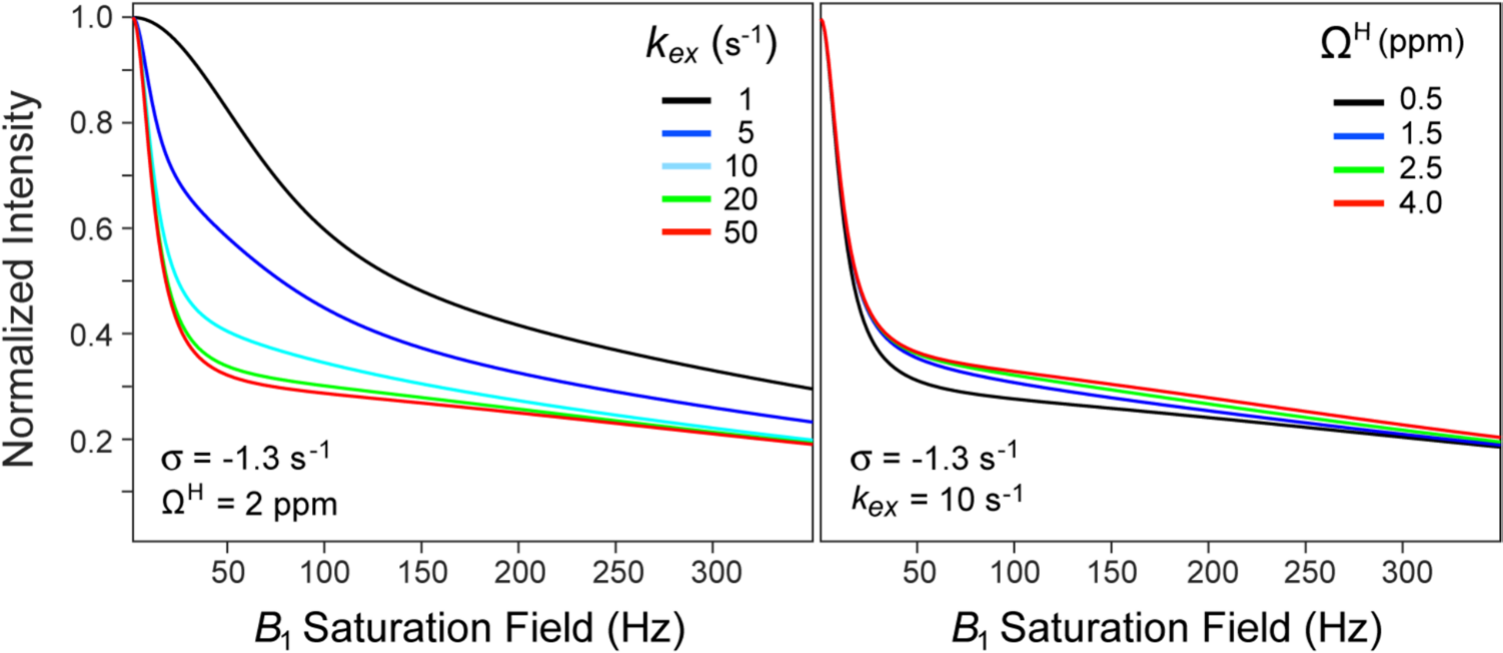
Simulations showing the decay of normalized signal intensities as a function of the saturation *B*_1_ field strength calculated using Eq. (1): (*Left panel*) for a range of *k*_*ex*_ values from 1 to 50 s^−1^, with σ fixed at -1.3 s^−1^, and Ω^H^ = 2 ppm. (*Right panel*) for a range of Ω^H^ values from 0.5 to 4 ppm, with σ = -1.3 s^−1^ and *k*_*ex*_ = 10 s^−1^. The *B*_1_ field (*ω*_*1*_) was applied at the frequency of water (Ω^W^ = 0) for a duration of 2.5 s. All simulations were performed for a spectrometer frequency of 600 MHz using the following set of typical parameters for Ileδ1 methyl groups of MSG at 5 °C: Ω^M^ = -4.3 ppm (from the carrier at 0); $R_{1}^{{\text{M}}}$ = 1.8 s^−1^; $R_{2}^{{\text{M}}}$ = 50 s^−1^; $R_{1}^{{\text{H}}}$ = 1.0 s^−1^; $R_{2}^{{\text{H}}}$ = 300 s^−1^; $R_{1}^{{\text{W}}}$ = 50 s^−1^; $R_{2}^{{\text{W}}}$ = 95 s^−1^; and σ_*R*_ = -2 σ = 2.6 s^−1^

Methyl ^1^H relaxation rates,$R_{2}^{{\text{M}}}$ and the ‘initial rate’ approximation to $R_{1}^{{\text{M}}}$, were measured for each methyl site of MSG by straightforward extensions of gradient selected (^1^H-^13^C)-HMQC experiments (average values < $R_{2}^{{\text{M}}}$ > = 49 s^−1^ and < $R_{1}^{{\text{M}}}$ > = 1.8 s^−1^ were obtained for 37 Ileδ methyl groups of {U-[^15^N, ^2^H]; Ileδ1-[^13^CH_3_]}-labeled MSG at 5 °C; 600 MHz) and fixed in the non-linear best-fits of methyl normalized intensity profiles as a function of saturation field strength, *ω*_1_, for three saturation frequency offsets from the water signal, -Ω^W^: 0 (on-resonance saturation), -60 and − -200 Hz. Multiple fits were performed using {σ; *k*_*ex*_; Ω^H^} as methyl site-specific variable parameters, and the rates {$R_{1}^{{\text{W}}}$;$R_{2}^{{\text{W}}}$} as global variable parameters on a grid of fixed and uniform (the same for all methyl sites) values of {$R_{1}^{{\text{H}}}$;$R_{2}^{{\text{H}}}$}. Selected examples of such best-fit intensity decay profiles obtained for Ileδ1 sites of MSG at 5 °C (600 MHz), performed with $R_{1}^{{\text{H}}}$ and $R_{2}^{{\text{H}}}$ set to 0.8 s^−1^ and 300 s^−1^, respectively, are shown in Fig. 3. Analysis of the results of such a grid-search showed that while the goodness-of-fit is very similar among individual minimizations, the best-fit σ values are remarkably robust to variations in {$R_{1}^{{\text{H}}}$;$R_{2}^{{\text{H}}}$}—in particular, the calculated maximal standard deviation of the mean σ, < σ > , is only 0.012 s^−1^ for a range of $R_{1}^{{\text{H}}}$ rates between 0.6 and 2.4 s^−1^ and a range of $R_{2}^{{\text{H}}}$ between 150 and 600 s^−1^. We verified that the same applies to individual values of *σ* for each methyl site. The best-fit values of *σ* for Ileδ1 sites of MSG at 5 °C varied from -0.6 to -3.5 s^−1^ (< σ > = -1.25 ± 0.63 s^−1^).

**Fig. 3:**
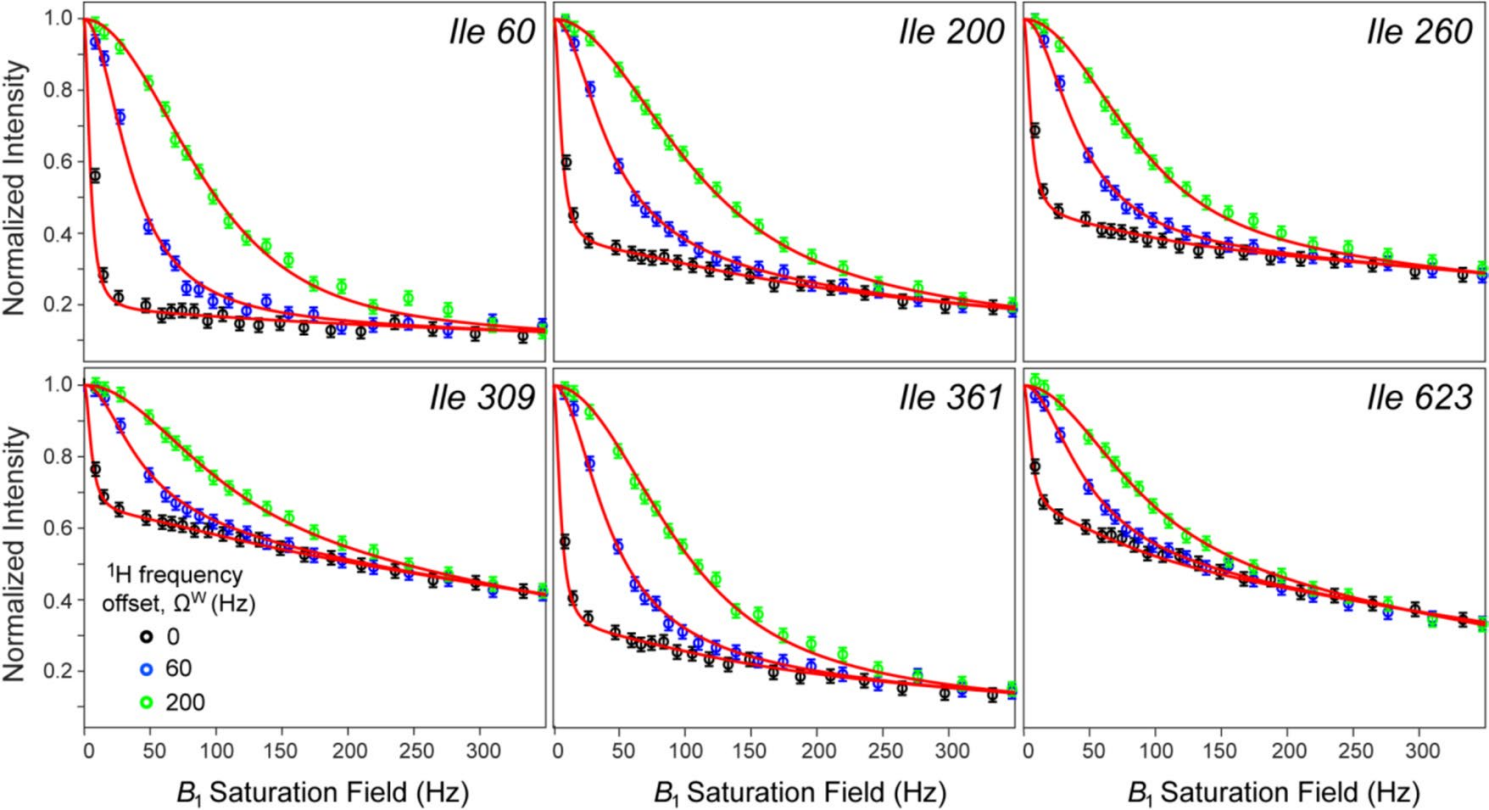
Selected examples of 2D cross-peak intensity decay curves obtained for the {U-[^15^N, ^2^H]; Ileδ1-[^13^CH_3_]}-labeled sample of MSG at 5 °C (τ_C_ ~ 90 ns) as a function of the saturation field strength, *ω*_1_. The data were obtained with the 2D dd-(^1^H-^13^C)-HMQC experiment preceded by a ^1^H CW saturation period with a duration of 2.5 s at or near the frequency of the water signal. The experimental data obtained with the ^1^H saturation performed on-resonance with the water signal are represented by open black circles, while the data obtained with ^1^H saturation frequency offset up-field of the water resonance by 60 and 200 Hz, are shown with blue and green open circles, respectively. The values fitted to the model in Eq. (1) at each frequency offset are shown with red solid curves. The fits were performed as described in the text using fixed and uniform values of $R_{1}^{{\text{H}}}$ = 0.8 s^−1^ and $R_{2}^{{\text{H}}}$ = 300 s^−1^. The data were acquired at 600 MHz; sample conditions are listed in Fig. 1

In principle, the cross-relaxation rates *σ* may be determined from a single decay profile, with water saturated on-resonance, albeit at the expense of other parameters of the model remaining ill-determined (especially, the offsets of labile protons from saturation frequency, Ω^H^). Recording of several decay profiles with different *B*_1_ field offsets, significantly improves the precision of the extracted *σ* rates. The choice of *B*_1_ field offsets is dictated by the steep dependence of the shape of decay profiles – particularly, the initial ‘drop-off’ of intensities – on the *B*_1_ field offsets (Fig. 3). We observed that the best results are achieved when the *B*_1_ field offsets, Ω^W^, are approximately uniformly distributed in the range, 0 ≤ Ω^W^ ≤ ~ 0.5*ω*_1,max_, where *ω*_1,max_ is the largest *B*_1_ field strength employed (typically, 350–400 Hz).

While the choice of $R_{2}^{{\text{H}}}$ in the fits to the model in Eq. (1), affects primarily the fitted values of Ω^H^ (*i.e.* the errors/variations in $R_{2}^{{\text{H}}}$ are absorbed mainly by Ω^H^), the choice of $R_{1}^{{\text{H}}}$ correlates and anti-correlates with the fitted *k*_*ex*_ and Ω^H^ values, respectively. As neither *k*_*ex*_ nor Ω^H^ can be meaningfully interpreted in the approximate model described by Eq. (1), we focus here exclusively on the cross-relaxation rate σ – the only parameter that is largely invariant to the choice of {$R_{1}^{{\text{H}}}$;$R_{2}^{{\text{H}}}$}. For reference, we note that in the fits where $R_{1}^{{\text{H}}}$ and $R_{2}^{{\text{H}}}$ are set to 0.8 s^−1^ and 300 s^−1^, respectively, *k*_*ex*_ for individual methyl sites varied between 1 and 30 s^−1^ (< *k*_*ex*_ > = 5.5 ± 4.3 s^−1^), while the values of Ω^H^ varied from + 0.3 to + 3.0 ppm from the chemical shift of water (< Ω^H^ > = + 1.8 ± 0.6 ppm from water). The optimized values of the rates $R_{1}^{{\text{W}}}$ and $R_{2}^{{\text{W}}}$ were 49.7 ± 1.2 s^−1^ and 95.6 ± 2.1 s^−1^, respectively, and report on the inhomogeneity of the applied *B*_1_ saturation field rather than the intrinsic relaxation rates of water ^1^H spins.

Figure 4A compares the values of σ obtained at 5 (black bars) and 30 °C (red bars). Although on average the values of |σ| scale approximately proportionately to the global tumbling times (< σ_5C_ > = -1.25 s^−1^ versus < *σ*_30C_ > = -0.69 s^−1^), several methyl sites show very similar *σ* rates at 5 and 30 °C (*e.g.* Ile^12^, Ile^42^; Fig. 4A) possibly reflecting different contributions of local side-chain motions at the two temperatures. To gain more confidence in the obtained cross-relaxation rates σ, we performed the measurements of methyl intensity decay profiles for Ileδ1 sites of MSG at 900 MHz (5 °C), and analyzed the data in the same manner with the model in Eq. (1). The correlation plot comparing the values of σ obtained at 600 and 900 MHz spectrometer fields (both at 5 °C) is shown in Fig. 4B. The values of σ at 900 MHz (*σ*_900_; *y*-axis) are consistently smaller by absolute magnitude (by 9% on average) than their counterparts obtained at 600 MHz (*σ*_600_; *x*-axis). This difference is much larger than the calculated contribution of the spectral density at 2*ω*_H_ frequency, *J*(2*ω*_H_), to *σ* for a molecule with a *τ*_C_ = 90 ns if only global molecular motion is considered, and may likewise reflect the role played by local motions in the interactions between methyl and labile proton spins.

**Fig. 4:**
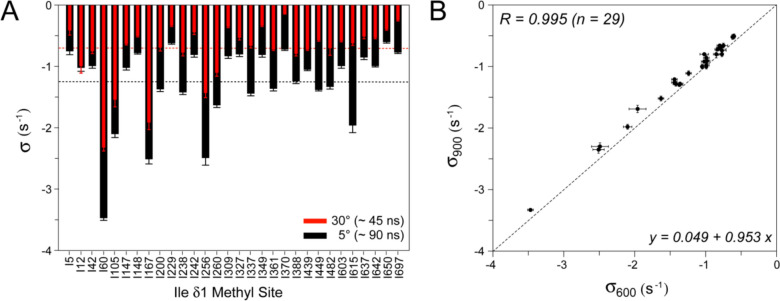
Comparison of σ values extracted for Ile δ1 methyl groups of {U-[^15^N, ^2^H]; Ile δ1-[^13^CH_3_]}-MSG using the model in Eq. (1) under different experimental conditions. **A** Plot comparing σ values obtained at 5 °C (wide black bars; τ_C_ ~ 90 ns) and 30 °C (narrow red bars; τ_C_ ~ 45 ns). Horizontal dashed lines correspond to the mean of all obtained σ values for each dataset. Both datasets were obtained at 600 MHz. **B** Correlation plot of σ values obtained at 600 MHz (*x*-axis) and 900 MHz (*y*-axis); both datasets were obtained at 5 °C. The parameters of linear regression for *n* = 29 methyl sites are shown on the plot. The diagonal dashed line corresponds to *y* = *x*

The largest values of |σ| obtained at either of the two or both temperatures (5 and 30 °C; Fig. 4A) are associated with Ile δ1 methyl sites that are located in close proximity to one or more labile, non-hydrogen-bonded protons in the three-dimensional structure of MSG (Howard et al. 2000) (pdb access code 1d8c). The distances to the nearest (located at ≤ 4.5 Å) labile, non-hydrogen-bonded protons in the structure of MSG for 6 methyl Ile δ1 sites with the largest |*σ*| rates (Fig. 4A) are summarized in Table 1. The shortest distance between methyl protons and the labile proton spin ‘H’, *r*_*hh*_ (estimated from the largest |*σ*| assuming the order parameter of the interaction *S*^2^ = 1, and *τ*_C_ = 90 ns for MSG at 5 °C, from the relationship, $\sigma = - \left( {3/10} \right)\left( {\mu_{0} /4\pi } \right)^{2} (\gamma_{H}^{4} \hbar {}^{2}S^{2} \tau_{C} /r_{{^{hh} }}^{6} )$is 4.0 Å, while the longest distance is 5.4 Å. We note that as the order parameters for methyl-side-chain (methyl ^1^H – labile ^1^H) interactions in a protein are quite unlikely to be close to unity, these values almost certainly represent upper bounds on inter-proton distances. It is also worth noting that the interpretation of the smallest cross-relaxation rates |σ| in terms of distances to neighboring labile ^1^H spins is prone to errors because labile protons whose exchange rates with water (*k*_*ex*_) are slowed down (e.g. due to inaccessibility to the solvent or hydrogen-bonding), may be present in close proximity to a given methyl Ileδ1 site. The proximity of such relatively slowly-exchanging labile protons to a given methyl site, however, will result in convex-shaped profiles (see Fig. 2, *left panel*) and will not be captured by the model in Eq. (1). Nevertheless, among 5 Ileδ1 methyl sites of MSG with the lowest |σ| rates, only one (Ile^5^) has a labile, non-hydrogen-bonded side-chain proton located at a distance of less than 5 Å (H^ζ3^ of Lys^17^ at 4.4 Å).

**Table 1:** Distances to the nearest (≤ 4.5 Å) labile, non-hydrogen-bonded protons for methyl Ile δ1 sites of MSG (Howard et al. 2000) with the largest |σ| rates

Ile δ1 Site	Labile ^1^H	Distance (Å)
Ile^60^	Arg^430^ guanidinium:	
	H^η2(b)^	2.9^(b)^
	H^η1(b)^	3.1^(b)^
	H^ε^	3.2
Ile^105^	Ser^384^ hydroxyl:	
	H^γ^	2.7
	Thr^102^ hydroxyl:	
	H^γ^	2.9
	Tyr^387^ hydroxyl:	
	H^η^	2.9
	Lys^421^ (NH_3_^+^)^(c)^	
	H^ζ^	4.0–4.5^(c)^
Ile^167^	Tyr^139^ hydroxyl:	
	H^η^	2.6
	Arg^171^ guanidinium:	
	H^η1(b)^	4.1^(b)^
Ile^256^	Tyr^139^ hydroxyl:	
	H^η^	4.0
	Arg^171^ guanidinium:	
	H^η1(b)^	2.9^(b)^
	H^η2(b)^	2.8^(b)^
Ile^260^	Ser^137^ hydroxyl:	
	H^γ^	4.0
	Ser^137^ backbone:	
	H^N^	4.3
	Arg^317^ guanidinium:	
	H^η2(b)^	4.3^(b)^
Ile^615^	Ser^618^ hydroxyl:	
	H^γ^	4.0

The distances calculated for each proton of a methyl group were (< *r*^−6^ >)^−1/6^-averaged. ^(b)^The shortest of the distances to the two η protons of guanidinium groups is indicated. ^(c)^The range of distances to the three ζ protons of NH_3_^+^ groups is specified

The application of pulsed field gradients to achieve quadrature detection in the indirect dimension of dd-(^1^H-^13^C)-HMQC experiments (Ahmed et al. 2024) used in this work, leads to a √2 loss in sensitivity compared to their amplitude-modulated analogues. For Ileδ1 methyl sites of MSG at 5 ºC, the major portion of this loss is recovered by the use of delayed decoupling. For methyl-labeled protein samples dissolved in H_2_O where the loss of a √2 is prohibitive, suppression of the water signal may be achieved by application of selective water pulses or selective excitation of methyl protons as developed for SOFAST-methyl-TROSY spectroscopy (Amero et al. 2009). Both these methods may be combined with the delayed decoupling scheme (Bolik-Coulon et al. 2023).

In summary, we show that deleterious decreases in methyl signal intensities are observed in selectively methyl-labeled protein samples of high-molecular-weight proteins dissolved in H_2_O, when water is saturated. These losses in sensitivity are traced to efficient cross-relaxation between methyl protons and proximal labile proton spins in the protein structure that are in fast exchange with (saturated) water. A phenomenological model describing exchange saturation transfer of magnetization from localized proton spins of water to labile groups in the protein structure that, in turn, efficiently cross-relax with methyl protons, allows for determination of cross-relaxation rates (σ) between methyl and labile protons and permits identification of methyl sites in close proximity to one or more labile groups in the protein structure.

## Data Availability

No datasets were generated or analysed during the current study.
